# Physicians and nurses professional relationship with criminal investigation in dealing with survivors of sexual abuse: a scoping review

**DOI:** 10.1186/s40352-023-00235-8

**Published:** 2023-08-24

**Authors:** Nuno Coelho, Anabela Neves, João Gregório

**Affiliations:** 1 Instituto de Polícia Judiciária e Ciências Criminais (Institute for Judiciary Police and Criminal Science), Loures, Portugal; 2 Center for Research in Biosciences and Health Technologies, CBIOS. Lusófona University, Lisbon, Portugal; 3 Health Sciences PhD Program, U Alcalá, Madrid, Spain; 4 Instituto Nacional de Medicina Legal e Ciências Forenses (National Institute of Forensic Medicine and Forensic Science), Coimbra, Portugal; 5 Medical and Forensic Office of North Lisbon Area, Lisbon, Portugal

**Keywords:** Sexual abuse, Interprofessional collaboration, Criminal investigation, Health professionals

## Abstract

Survivors of sexual abuse and their families seek help from criminal law enforcement agencies and health professionals to obtain justice and health care. Many communities have implemented multi-professional collaborative models so that the victim’s well-being is assured and the truth is established. However, there is a general lack of evidence on how to best articulate these teams with the healthcare professionals caring for the survivors.

Therefore, this Scoping Review was conducted in order to analyze and to map the barriers and facilitators of the relationship between health professionals and the criminal investigation team in the care of survivors of sexual abuse. The methodology proposed by the Joanna Briggs Institute for Scoping Reviews was used, and the Bronstein five dimension model of interprofessional collaboration served as the basis for the analysis of barriers and facilitators. Quantitative, qualitative and mixed studies, primary and secondary sources, text and opinion documents were included. Content analysis was performed on the main findings of the collected studies. Twelve articles were identified and analyzed. Collaboration, communication, hierarchy, skills, confidentiality, and leadership emerged as key themes. Multidisciplinary Sexual Assault Nurse Examiner (SANE) and Sexual Assault Response Teams (SARTs) were implemented to coordinate care, but conflicting goals and values among professionals posed challenges. Communication failures and inadequate information sharing hindered collaboration. Neutral leaders who coordinate teams, minimize groupthink, and improve decision-making were found to be valuable. Engaging across disciplinary boundaries and addressing power dynamics were challenging but could be addressed through facilitation and conflict resolution. This review highlights the importance of effective collaboration and interaction within teams and with other professionals in the care of sexual abuse survivors.

**Supplementary Information:**

The online version contains supplementary material available at 10.1186/s40352-023-00235-8.

## Background

Sexual abuse crimes adapt and evolve following the development of social norms and standards, resulting in high mental health and social consequences, and high economic costs (World Health Organization & Pan American Health Organization, 2015). In the United States of America alone, the economic burden of sexual abuse is estimated at nearly 3.1 billion USD over the lifetime of the survivors, with 1 billion USD being fully supported by public budgets (Peterson, DeGue, Florence & Lokey, 2017). This estimate includes medical costs, lost work productivity among survivors and offenders, in criminal justice activities and other costs, including loss or damage to the victim’s property.

Many of these survivors and their families turn to the criminal investigation police and health professionals for justice and health care (Greeson, Watling Neal & Campbell, 2019; McMahon and Schwartz, 2011; Moylan et al., 2017). However, the way hospital institutions are organizationally structured, challenges their management process (Vendemiatti et al., 2010), as well as the collaborative work between the parties involved in assisting these survivors and their families (Nero, 2008). For this reason, the needs of different communities led to the implementation of networks of multi-professional and collaborative models in order to respond to these victims, known as Sexual Assault Response Teams (SARTs)(Moylan & Lindhorst, 2015) or Sexual Assault Referral Centres (SARCs)(Greeson & Campbell, 2013).

However, the implementation of these models has not always been peaceful, due to their complexity and the professional barriers experienced (Moylan & Lindhorst, 2015). The traditional relationship between police and health professionals has often been one of conflict rather than collaboration in medico-legal cases (Kären M. & Hess, 2009). Since the 1970s, the relational constraints between police and nurses have been discussed (Moylan & Lindhorst, 2015). Only one-third of the Sexual Assault Nurses Examiners (SANEs) interviewed described their relationship with the police as positive, while one-third indicated that they had both good and bad experiences with them (Maier, 2012). Criminal investigation teams often have the perception that physicians and nurses obstruct justice while providing care to the victim and/or patient. Similarly, doctors and nurses perceive the police as invasive to patients’ privacy or as a pressure factor in the delivery of care during the investigation process (Maier, 2012).

The World Health Organization (WHO) has long recognized the importance of interprofessional collaborative work to ensure the successful delivery of health care (World Health Organization [WHO], 1978). Interprofessional collaboration is defined as a relationship in which at least two professionals work together towards common goals (Green & Johnson, 2015). Effective planning, decision making, and responding to shared problems and/or needs are part of that relationship (Idol & West, 1991). The possibility of creating solutions as a team to improve service delivery, which would be difficult to achieve individually, is the true potential of the interprofessional relationship (Bronstein, 2003; Petri, 2010).

Bringing together professionals from various professions and/or institutions to solve problems collaboratively should always result in more effective solutions (Opie, 2008; Sims et al., 2015). There is considerable evidence of how health professionals actively contribute to interprofessional collaborations, as well as how their differing worldviews and experiences mold the success of those collaborations (Schot, Tummers & Noordegraaf, 2020). Evidence shows that team coordinated efforts can lead to a better communication between professionals in the care given to victims of sexual abuse, shorter waiting time in hospitals for the victims, as well as more effective evidence collection (McMahon & Schwartz, 2011; Moylan & Lindhorst, 2015). However, studies on interprofessional work show that interprofessional teams can be compromised by lack of institutional support, lack of training in performing interprofessional work and lack of trust among team members (Greeson & Campbell, 2013; Kelty et al., 2013; Moylan & Lindhorst, 2015). To reinforce this idea, a meta-analysis conducted in 2003 (De Dreu & Weingart, 2003), identified several factors that can hinder the performance of interprofessional teams. Among them are interpersonal conflicts arising from personality conflicts, task conflicts due to disagreements about the objective of the teamwork and finally work process conflicts, or barriers arising from different opinions about how the teamwork should be carried out. Hierarchical concerns within institutions and competition for professional skills can also be reasons inducing lack of trust (Nugus, Greenfield, Travaglia, Westbrook, & Braithwaite, 2010).

Although there is now evidence of the effectiveness of interprofessional teams, especially in the healthcare field (Maier, 2012; Shin et al., 2021), the knowledge about the relations between teams from different organizations, such as the relation of healthcare teams with criminal police agencies, is still scarce. To bridge this gap, it is pertinent to conduct a scoping review aiming at colleting the evidence on the known barriers and best practices that enhance the performance of interprofessional teams that deal with sexual abuse survivors. To guide this process, this review was informed by the following research questions: What is the evidence on the professional relationship between health professionals (nursing and medicine) and criminal investigation teams, when addressing survivors of sexual abuse (number of studies and their characteristics)? What is known about the professional relationship between health professionals (nursing and medicine) teams with criminal investigation in dealing with survivors of sexual abuse (what does it mean, what barriers and facilitators influence this relationship)?

## Methodology

To achieve the aim of this literature review, a preliminary search was conducted in the JBI Evidence Synthesis (Munn, 2020), Cochrane Library and MEDLINE (Pubmed) databases. No current systematic reviews or scoping reviews on the professional relationship of health professionals (nurses and physicians) with the criminal investigation team dealing with survivors of sexual abuse were identified. Therefore, it was decided to conduct a scoping review, guided by the methodology proposed by the Joanna Briggs Institute for Scoping Reviews (Peters et al., 2015), with the objective of analyzing the known barriers and best practices in the interprofessional collaboration between physicians, nurses and the criminal investigation team of sexual abuse survivors.

### Conceptual framework

**Fig. 1:**
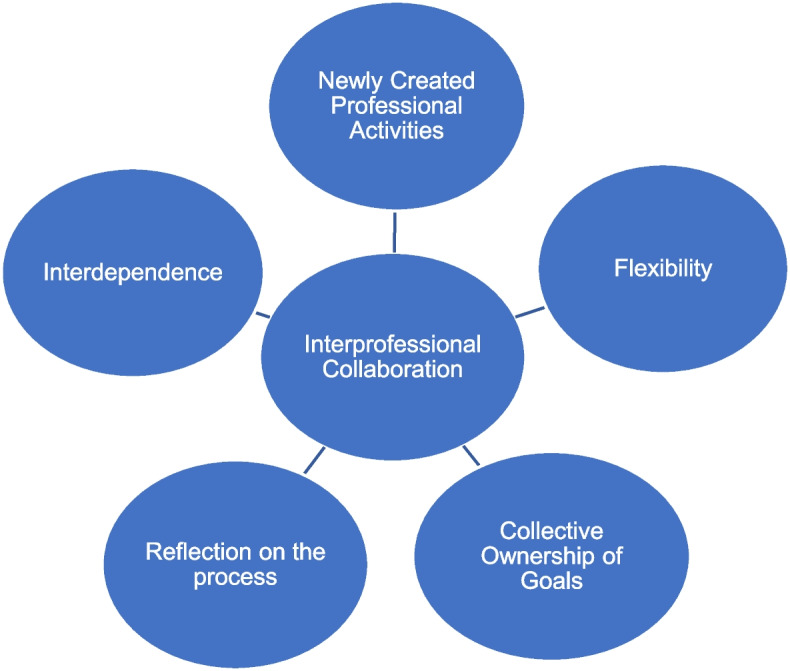
Adapted by the authors from the elements of a model of interdisciplinary collaboration (Bronstein, 2003)

The conceptual framework for data collection was based on the two-part model developed by Bronstein (Bronstein, 2003) (Fig. 1).

This model was chosen because it presents a description of the components of the ideal collaboration between health professionals: Interdependence, Newly created professional activities, Flexibility, Collective ownership of goals and outcomes, and Reflection on the process. Interdependence, refers to the existing interactions with other professionals to accomplish goals and tasks (Bronstein, 2002, 2003; Patterson & Pennefather, 2015); Newly created professional activities define the collaborative acts, programs and organizations allowing the fulfillment of goals that would not be achieved individually; Flexibility, although related, is distinct from interdependence, referring to the deliberate blurring of professional roles; Collective ownership of goals and outcomes demonstrates shared responsibility throughout the process of goal achievement, which includes joint planning, definition, and achievement, and is considered a facilitator of collaboration; Reflection on the process refers to the evaluation of those involved in the working process and the efforts expended. Although this conceptual framework was initially developed to assess the collaboration between social workers and other professionals, it meets our needs to understand the processes of interprofessional collaboration. As structural parts of professional collaboration, this model predicts Professional Role, Structural Features, Personal Features, and Collaboration History.

### Search strategy

The search strategy included the electronic databases CINAHL Complete, MEDLINE Complete, MedicLatina, Nursing & Allied Health Collection: Comprehensive, with Full Text, via the EBSCOhost platform, and a free Google Scholar search for gray literature, using a Boolean phrase combining the descriptors for the acronym PCC (see Table 1 for more detail):

**Table 1:** Search strategy and limiters applied per database and the respective search results per database

Database: MEDLINE (via EBSCOhost) Results: 4634 Search strategy (January 2022)	
(“nurs*“[Tx]) OR “health professional “[Tx]) OR “Physicians“[Tx]) OR “Doctor“[Tx]) AND “police”[Tx]) OR “”child sexual abuse””[Tx]) OR “”sexual abuse”“[Tx]) OR “rape“[Tx]) OR “”sexual offense””[Tx]) OR “”sexual violence”“[Tx]) AND “interprofessional“[Tx]) OR “multidisciplinary“[Tx]) OR “collaboration“[Tx]) OR “relation“[Tx]) OR “”collaboration interprofessional”“[Tx])	
Database: CINAHL Complete (via EBSCOhost) Results: 2879 Search strategy (January 2022)	
(“nurs*“[Tx]) OR “health professional“[Tx]) OR “Physicians“[Tx]) OR “Doctor“[Tx]) AND “police”[Tx]) OR “”child sexual abuse””[Tx]) OR “”sexual abuse”“[Tx]) OR “rape“[Tx]) OR “”sexual offense””[Tx]) OR “”sexual violence”“[Tx]) AND “interprofessional“[Tx]) OR “multidisciplinary“[Tx]) OR “collaboration“[Tx]) OR “relation“[Tx]) OR “”collaboration interprofessional”“[Tx])	
Database: MedicLatina, Nursing & Allied Health Collection (via EBSCOhost) Results: 1427 Search strategy (January 2022)	
(“nurs*“[Tx]) OR “health professional“[Tx]) OR “Physicians“[Tx]) OR “Doctor“[Tx]) AND “police”[Tx]) OR “”child sexual abuse””[Tx]) OR “”sexual abuse”“[Tx]) OR “rape“[Tx]) OR “”sexual offense””[Tx]) OR “”sexual violence”“[Tx]) AND “interprofessional“[Tx]) OR “multidisciplinary“[Tx]) OR “collaboration“[Tx]) OR “relation“[Tx]) OR “”collaboration interprofessional”“[Tx])	
Database: Google Scholar Results: 34 Search strategy (January 2022)	
(nurs* OR "health professional" OR Physicians OR Doctor) AND (police OR "criminal investigation" OR criminal) AND ("child sexual abuse" or "sexual abuse" or rape or "sexual offense" or "sexual violence") AND (interprofessional or multidisplinary or collaboration or relation or "collaboration interprofessional")

- **Participants** were physicians, nurses and criminal investigators;
- The **Concept** was the professional relationship between them;
- The **Context** was the care for victims or survivors of sexual abuse in a health care setting.

We adopted the WHO definition of sexual abuse as (World Health Organization [WHO], 2014):“any sexual act or attempt to procure a sexual act, unwelcome comments or innuendos concerning sex, acts aimed at trafficking or directly directed at a person’s sexuality, performed by means of coercion, by any individual, regardless of his or her relationship to the victim, in any situation, including at home and at work”, and the definition of child sexual abuse as “contacts between a child and an adult or other person significantly older or in a position of power or control over the child, where the child is being used for sexual stimulation by the adult or other person” (Acuff et al., 1999).

### Inclusion criteria

Quantitative, qualitative and mixed studies, primary and secondary sources, were included. Text and opinion papers were also considered in the review. Only studies written in English, Spanish, and Portuguese, regardless of the year of publication, were considered for this review. Articles reporting situations of sexual abuse in armed conflict were excluded.

After collecting the references, titles and abstracts were screened by the main author, and those with agreement with the PCC were selected; then two authors read the studies that met the inclusion criteria and their full texts obtained to verify the suitability of the articles according to the inclusion criteria. Finally, the reference list of all studies selected for the critical appraisal was further reviewed for additional studies.

### Data extraction

One researcher (NC) extracted and two researchers (NC and JG) verified the data using an MS® Excel spreadsheet developed by the research team, aligned with the review aim and research questions. Whenever the reviewers had doubts about the relevance of a study during the reading of the abstract, they obtained the full article. The studies identified from reference lists were also assessed for relevance based on their title and abstract.

Content analysis with a deductive approach was conducted on the data collected from the literature review. Content analysis is a systematic research method used to analyze textual data, in order to uncover patterns, themes, and categories (Finfgeld-Connett, 2014). The deductive approach used the dimensions of Bronstein’s model to identify relevant units of analysis that could be classified as a barrier or facilitator for each dimension. Afterwards, two authors (NC and JG) reviewed the findings and assigned codes independently. These initial codes were then reviewed and discrepancies were discussed. Finally, the codes were grouped together in broader themes.

## Results

**Fig. 2:**
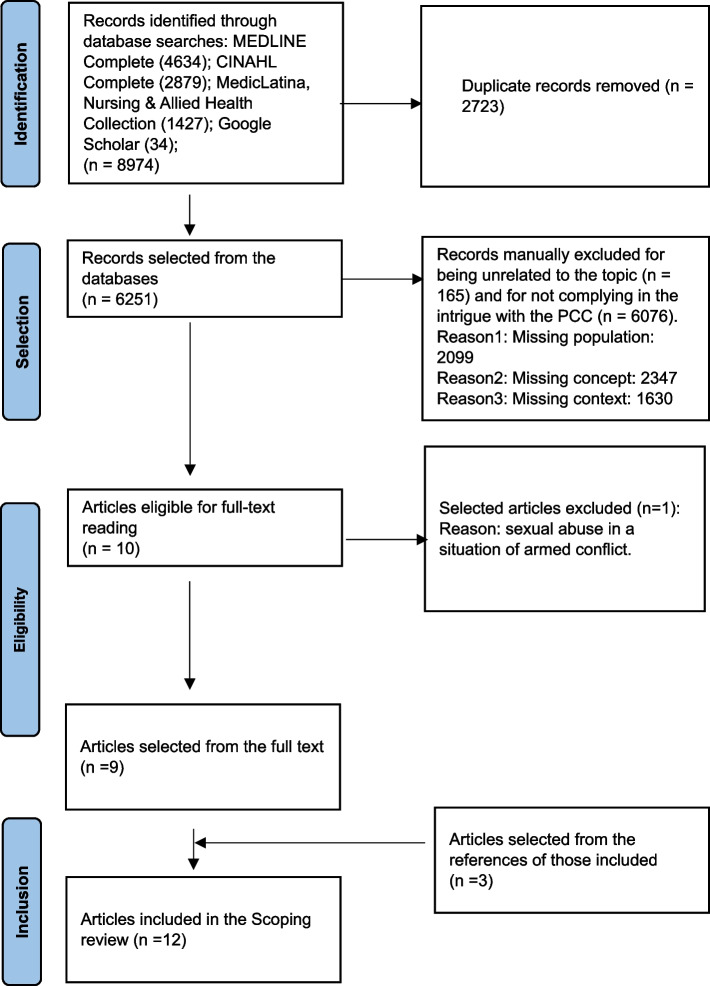
PRISMA Flowchart (adapted) of the study selection process

As presented in Fig. 2, the search identified 8974 potentially relevant studies. Of these, 2723 were excluded for being duplicates; 165 were excluded for being unrelated to the topic and 6076 for not fully complying with the PCC. Of the remaining ten references, one was excluded for reporting data obtained in armed conflict. From the nine final articles, the reference lists were read and three more papers were added. In the end, 12 articles were included in this review (Table 2).

**Table 2:** Research methods of the studies (*n* = 12)

Research method	N (%)
Qualitative	8 (66%)
Mixed (quantitative and qualitative)	2 (17%)
Case study	2 (17%)

Most studies used qualitative methods (*n* = 8) (Campbell, Greeson, Bybee, & Fehler-Cabral, 2012; Cole, 2011; Herbert et al., 2021a; Kelty et al., 2013; Moylan and Lindhorst, 2015; Moylan et al., 2017; Tien et al., 2017; Wegrzyn et al., 2021); while the remaining used mixed methods (*n* = 2) (Adams & Hulton, 2018; Campbell et al., 2011), or a case study approach (*n* = 2) (Greeson et al., 2019). More than half of the included studies (Adams & Hulton, 2018; Greeson et al., 2019; Herbert et al., 2021; Moylan & Lindhorst, 2015; Moylan et al., 2017; Tien et al., 2017; Wegrzyn et al., 2021)(*n* = 8) were carried out between 2015 and 2021.

The included articles mostly reported research efforts conducted in the United States ( Adams and Hulton, 2018; Campbell et al., 2011; Campbell et al., 2012; Cole, 2011; Moylan and Lindhorst, 2015; Moylan et al., 2017; Wegrzyn et al., 2021)(*n* = 8), followed by Australia (Greeson et al., 2019; Herbert et al., 2021; Kelty et al., 2013) ) (*n* = 3), and Taiwan (Tien et al., 2017)(*n* = 1). Among the selected studies, there was a notable absence of European studies.

The majority of the studies evaluated the general collaboration between team members (Adams & Hulton, 2018; Campbell et al., 2011, 2012; Greeson et al., 2019; Herbert et al., 2021; Moylan & Lindhorst, 2015; Moylan et al., 2017; Tien et al., 2017; Wegrzyn et al., 2021) *n* = 10). The remaining focused on specific dimensions, specifically communication within the team (Kelty et al., 2013) (*n* = 1), and perceptions of victims of sexual abuse’s advocates about the revictimization of sexual abuse victims by the police and the health system (Cole, 2011) (*n* = 1).

Considering the five dimensions of the analyzed model, themes related to Collaboration, Communication and Hierarchy stood out, as barriers in the five dimensions. Regarding the facilitators, the themes related to Collaboration, Leadership, and Relationships stood out (Tables 3 and 4). In the following paragraphs, an analysis of the barriers and facilitators for each of the Bronstein’s framework dimensions is provided. For a detailed description, see Table S1 (Supplementary material).

**Table 3:** Content analysis of barriers classified according to the five dimensions of Bronstein’s model of interprofessional collaboration

Themes	Interdependence	Newly created professional activities	Flexibility	Collective ownership of objectives	Reflection on the process
**Collaboration**	• Inequality in collaboration • Isolated work. • Different level of collaboration. • Lack of interest in collaborating.	• Different work practices and philosophies.	• Lack of professional autonomy. • Undervaluation and interference in the work of others. • Divergent practices and points of view.	• Lack of common goals. • Low satisfaction within the team. • Conflicting objectives within the team. • Not working full time. • Different philosophies, goals, and values.	• Rare meetings about sexual assault cases.
**Skills**			• Function limits • Role confusion		
**Communication**	• Tension in communication. • Omissive communication. • Divergent opinions. • Divergent concepts.	• “Silo Effect”.	• Misunderstanding of the functions.	• Lack of face-to-face meetings.	• No information outside the team.
**Confidentiality**	• Different confidentiality policies.		• Different obligations and rules regarding confidentiality.		
**Hierarchy**	• Differences in status. • Influence of older people on younger people.	• Hindered coordination by lack of understanding an interprofessional model.	• “turf wars”	• Power imbalances and difficulty in developing common goals.	
**Organizational**	• Confusion of boundaries between organizations. • Group rotation. • Different organizational support.	• Lack of time and financing. • Differences in valuation between organizations		• Blurred boundaries between systems. • Lack of resources and time. • Different goals in different organizations. • Shift work.	• Time and space to meet with other professionals.
**Relationship**	• Groupthink • Social conformity • Tunnel vision • Conflicts between disciplines • Divergent working practices • HP’s Perceptions of undervalued knowledge • Lack of interaction between professionals • Too much centralization		• Conflicts resulting from role negotiation. • Lack of respect for the roles of others.	• Lack of trust among the team. • Lack of interprofessional connection.

**Table 4:** Content analysis of facilitators classified according to the five dimensions of Bronstein’s model of interprofessional collaboration

Themes	Interdependence	Newly created professional activities	Flexibility	Collective ownership of objectives	Reflection on the process
**Collaboration**	• Team support. • Mutual commitment • The perception of effective collaboration.	• Informal working groups. • Foster networking and teamwork abilities.	• Establish roles and responsibilities.	• Mutually agreed (HP and Police) common protocols. • Common goals for the group.	• Analysis of cases and protocols. • Reflection on the functioning of teams.
**Skills**	• Recognition of the other professional’s roles and responsibilities. • Competence of SANEs. • Problem solving strategies				
**Communication**	• Protocols of action. • Information sharing. • Clear procedures for team communication.		• Building mutual knowledge of professional roles.		• Clear disclosure of team’s decisions • Disclosure of information • Regular meetings of the coordination team.
**Leadership**	• Well-trained group leader. • Team leader as the facilitator. • Clear hierarchy	• Coordination in the SARTs.	• Clear definition of roles by the coordinator.	• Neutral team leader. • Leadership can guide collaboration and assist in communication and conflict resolution.	• Motivation of the leader • Facilitating team leader.
**Organizational**		• SANE Implementation. • Training of SANE Nurses. • SANE Teams.		• Organizational relationships. • Coordination of care services by SARTs.	• Organizational support. • Frequent reassessment of team functioning. • Developing infrastructures.
**Relationship**	• Respect and trust. • Personal knowledge of the team. • Socialization.	• Training by SANE. • Forming multidisciplinary informal working groups.		• Interprofessional models with well established activities and responsibilities (e.g. SART).	• Multidisciplinary training. • Promotion of relationships with all stakeholders. • Building trust.

### Interdependency

In this dimension, related to the Communication theme we found that some of the barriers for interprofessional work are omissive communication patterns (Kelty et al., 2013), divergent opinions (Moylan et al., 2015), divergent understanding of the concept of victim among health professionals and police, tensions and conflicts between disciplines and high rotation/turnover of professionals within the team (Greeson et al.,^2019^; Wegrzyn et al., 2021).

Related to confidentiality, different confidentiality policies were referred by health professionals. Isolated work, when traditional services work in isolation from each other and the linkage and coordination between systems is often weak, different level of collaboration and a lack of interest in collaborating were found in the theme of collaboration (Tien et al., 2017). Regarding relationships, conflicts between physicians and nurses and different policies on victim confidentiality led to conflicts between health professionals and the police (Cole, 2011); Errors in decision-making, due to groupthink, social conformity, tunnel vision and context bias were also found (Kelty et al., 2013).

To address some of the barriers in Interdependency, Communication is also key, with the creation of protocols of action and clear procedures for team communication as the main facilitators. A well trained leader and a clear hierarchy were also found as facilitators (Kelty et al., 2013). Other topics emerged as facilitators such as perception of interprofessional collaboration among police, physicians and nurses, (Tien et al., 2017); the ability to know the email of another professional (Kelty et al., 2013); information sharing between institutions (Greeson et al., 2019), as well as the construction of interprofessional relationships of trust (Wegrzyn et al., 2021) and the use of problem-solving strategies (Moylan et al., 2015).

### Newly created professional activities

The main barriers for the dimension of “Newly Created Professional Activities” were related to Hierarchy and Organizational. Different practices and philosophies (Adams & Hulton, 2018; Tien et al., 2017), as well as the differences in valuation between organizations (Greeson et al., 2019) hinders team work. Lack of time, lack of funding for training (Herbert et al., 2021; Wegrzyn et al., 2021) and the existence of a “silo effect” between the forensic teams and the police are also mentioned (Kelty et al., 2013).

Regarding facilitators in this dimension, the implementation of SANE programs and SARTs (Campbell et al., 2012), stood out as did the aspect of good coordination across SARTs that leads to more efficient evidence gathering (Tien et al., 2017). Fostering networking and teamwork abilities by the creation of informal working groups of interdisciplinary practices from other areas of intervention (Kelty et al., 2013) were also found as important facilitators in this dimension.

### Flexibility

In the dimension of Flexibility, barriers for interprofessional work between health professionals and police, were mainly in the theme of Collaboration. Divergent practices and points of view, lack of professional autonomy and interference in the work, have emerged as barriers to interprofessional work (Campbell et al., 2011; Greeson et al., 2019; Kelty et al., 2013; Moylan et al., 2015, 2017; Wegrzyn et al., 2021). These barriers also reflect the healthcare professionals’ perception of greater appreciation for evidence collection rather than victim support (Tien et al., 2017). Role confusion, lack of clear boundaries between the roles of various team members and different obligations and rules on confidentiality and information sharing (Moylan & Lindhorst, 2015), as well as “turf wars”(Greeson et al., 2019), were the remaining barriers found.

In this dimension, Leadership and Collaboration emerged as the most important themes, supported by the need of a clear definition of roles and responsibilities established by the team coordinator. Rules, roles and procedures can help to increase articulation and collaboration within teams (Wegrzyn et al., 2021) which by themselves are important to build mutual knowledge of each team members’ roles (Herbert et al., 2021).

### Collective ownership of objectives

In this dimension, Collaboration and Organizational are notable barriers for interprofessional work. Shift work, low work satisfaction, conflicting goals and objectives in different organizations were the most significant barriers. Shift work hinders the ability of teams to work together (Tien et al., 2017). Moreover, conflicts, power imbalances and difficulty in developing common goals among professionals (Wegrzyn et al., 2021), also emerged as barriers.

The mains facilitators were related to Leadership, with a neutral team leader/coordinator, who promotes articulation between police and health professionals (Moylan et al., 2017), emerging as relevant finding. Mutually agreed common goals for the group (Kelty et al., 2013) and using well tried and tested interprofessional models (e.g. SARTs) are also valuable strategies to facilitate the collective ownership of objectives.

### Reflection on the process

In this dimension, not having dedicated time and space for team meetings (Tien et al., 2017), was found as the major Organizational barrier. This may stem from the fact that meetings between institutions and professional were more common in homicides than in cases of sexual assault and that only those present at the meetings have access to information from the group discussions (Kelty et al., 2013).

Thus, naturally, the main facilitator were regular coordination meetings for each case to allow the team to communicate with each other (Moylan & Lindhorst, 2015; Moylan et al., 2017; Tien et al., 2017; Wegrzyn et al., 2021). Those meetings should be carried out with a well-trained leader, with organizational support (Kelty et al., 2013; Moylan & Lindhorst, 2015), with ongoing training, and reflection on the functioning of teams (Herbert et al., 2021).

## Discussion

This scoping review aimed to analyze the professional relationship between physicians and nurses with criminal investigation teams in the care of survivors of sexual abuse, as well as the barriers and facilitators influencing this relationship. Twelve articles were identified and analyzed in this scoping review. Barriers and facilitators of Bronstein’s five dimension model aligned along themes such as Collaboration, Communication, Hierarchy, Skills, Confidentiality and Leadership.

Due to the necessity to find a better articulation between professionals, multidisciplinary SANE and/or SART teams were implemented to coordinate care for victims of sexual abuse (Buschur, 2012; Wegrzyn et al., 2021). The existence of these teams has contributed to most of the evidence regarding multidisciplinary work in the care for survivors of sexual abuse. However, the dual goals of SART, which may sometimes be in conflict, and the philosophies and values markedly different of the professionals who participate in the SART (e.g. nurses and police officers) may pose an even greater challenge to multidisciplinary team collaboration than what can be found in multidisciplinary healthcare teams. Another major challenge refers to failures in communication, which can cause misunderstandings in the way information is received or passed on to the teams (Adams & Hulton, 2018). Studies have shown the importance of adequate information sharing and transmission, as fundamental for interprofessional collaboration (D’Amour, Goulet, Labadie, San Martín-Rodriguez, & Pineault, 2008; San Martin-Rodriguez, Beaulieu, D’Amour, & Ferrada-Videla, 2005).

The benefits of collaborative teams, increases the effectiveness of the whole interventional process with the survivor of sexual abuse. Understanding the team’s structure is an important factor for the team’s success due to their inherent network functioning (Greeson et al., 2019). Trust and mutual respect are essential, but it is also important to acknowledge that teams may experience conflict and tension. The domains of disciplinary knowledge tend to reveal themselves as a barrier by overlapping the interprofessional work (Ellingson, 2002). Some of the articles mentioned other professionals involved in dealing with victims of sexual abuse, such as teachers, social workers (Hicks & Tite, 1998) and advocates (professionals trained to support victims by providing information, emotional support and helping them with the bureaucracy) (Maier, 2008). However, multidisciplinary teams in healthcare are primarily concerned with promoting patient health. The importance of identifying effective multi-organisational interactions is critical as many of these staff will have divergent working practices and views on their role. The structural characteristics, such as the degree of bonding in the team or how relationships are shared evenly versus unevenly across the team, may be associated with improved team functioning, whereas in the absence of such strong bonds conflicts can happen. It can also lead to organizations giving up on collaborating altogether rather than continuing work to find a shared solution. Appreciating other people’s work may be necessary for team members to develop worthwhile coordination strategies. A strong leadership that minimizes groupthink, tunnel vision and improves decision-making (Kelty et al., 2013), will be key to coordinate such a diverse team. An important finding of this review is the figure of a neutral leader, someone who is neither health professional nor police investigator, to coordinate these teams.

The collaboration and interaction within teams and with other teams is fundamental, because those with less experience in real cases and less knowledge about the relationship between abuse and behaviour are likely to be the first point of contact for reports at any given time (Hicks & Tite, 1998). The need to carry out meetings with staff from other institutions, understanding the roles and responsibilities of other team members can be a facilitator of collaboration for a new team. This facilitator is supported by Edwards (2011), where he highlights the importance of building interprofessional knowledge during team meetings. The ability to put a name to an email, the personal contact with someone from another institution or discipline, facilitates the care to the survivor. Having a real point of contact for discussion, when something seems “potentially wrong”, leads to a more comprehensive understanding of the complexities involved in specific cases (Kelty et al., 2013).

Engaging across disciplinary boundaries may seem to be the most unsurpassable barrier, despite the unifying discourse on teamwork (Moylan et al., 2017). This may be due to the coexistence of both competitive and collaborative power in health services, being exercised in the dimensions of decision-making, care provision and assessment of care provision. Collaborative power involves interdependent participation, through the differentiation of roles and decision-making (Nugus et al., 2010). Evidence suggests that the solution to this problem could be the creation of a leader with the skills to facilitate conversations in interprofessional meetings, as well as to discuss deeply the conflicts as they arise, in order to solve them and find structural elements to support the involvement process (Moylan & Lindhorst, 2015).

### Limitation of scoping review

As is the norm with any review, the inclusion criteria may have influenced the results here presented. In this review, we decided to include only articles published in English, Spanish and Portuguese. Thus, articles published in other languages that could have been important for this scoping review were not included. The very significant differences between the initial number of papers collected and the final number of articles included happened because most papers did not focus simultaneously on the three components of the PCC, that is the professional and/or institutional relationship between physicians and nurses with criminal investigators, in the approach to survivors of sexual abuse. Also, no quality assessment was performed, potentially affecting the overall reliability and validity of the conclusions.

## Conclusion

The success of an effective response to provide comprehensive care and establish the truth in dealing with survivors of sexual abuse is closely related to the professional relationship of physicians and nurses with criminal investigation. Although there is some evidence of collaborative working between these teams, there is little information on the relationship between these health professionals and the criminal investigation in the specific context of sexual abuse.

Taking into account the current social responses, this scoping review finds that to improve the interprofessional relationship teams and managers should focus on three aspects - Leadership, Communication and Collaboration. The leadership, the creation of protocols and guidelines for action at the organisational level may be useful to guide nurses and physicians in the development of professional relationships with police officers.

According to the data collected in the research, this scoping identified some factors that require further investigation in the future, where SART teams, which include physicians and nurses, are not yet part of the response of the health and justice system. It highlights the need to create and apply methods to evaluate the intervention of these teams dealing with survivors of sexual abuse, the valorization of collaborative work and a clear delimitation of the responsibilities of each organization. It is also important to carry out further pre and post intervention studies, assessing the needs of each team in their relationship and its impact on the victim, society and justice system.

### Supplementary Information

**Additional file 1.**

## Data Availability

The patient level data that support the findings of this study are owned by the National Department of Health and the private health care providers who participated in the study, and so are not publicly available. Data are however available from the authors upon reasonable request and with permission of the National Department of Health (South Africa) and private health care providers.
