# Supplementary material for: Physicians and nurses professional relationship with criminal investigation in dealing with survivors of sexual abuse: a scoping review
Source: Health Justice. 2023 Aug 24;11:33. doi: 10.1186/s40352-023-00235-8 (PMC10464451; doi:10.1186/s40352-023-00235-8)
Supplement: Supplementary file 1 — Additional file 1. [file 40352_2023_235_MOESM1_ESM.docx]

Table S1 - Detailed list of Barriers and facilitators found in the original papers
